# Investigating the genetic architecture of general and specific psychopathology in adolescence

**DOI:** 10.1038/s41398-018-0204-9

**Published:** 2018-08-08

**Authors:** Hannah J. Jones, Jon Heron, Gemma Hammerton, Jan Stochl, Peter B. Jones, Mary Cannon, George Davey Smith, Peter Holmans, Glyn Lewis, David E. J. Linden, Michael C. O’Donovan, Michael J. Owen, James Walters, Stanley Zammit, the 23 and Me Research Team

**Affiliations:** 1 Centre for Academic Mental Health, Population Health Sciences, Bristol Medical School, University of Bristol, Bristol, UK; 2 Medical Research Council (MRC) Integrative Epidemiology Unit (IEU), University of Bristol, Bristol, UK; 3 NIHR Biomedical Research Centre, University Hospitals Bristol NHS Foundation Trust, University of Bristol, Bristol, UK; 4 Department of Psychiatry, University of Cambridge, Cambridge, UK; 5 Department of Psychiatry, Royal College of Surgeons in Ireland, Dublin, Ireland; 6 MRC Centre for Neuropsychiatric Genetics and Genomics, Division of Psychological Medicine and Clinical Neurosciences, Cardiff University, Cardiff, UK; 7 Division of Psychiatry, University College London, London, UK; 8 23andMe, Inc., Mountain View, CA USA

## Abstract

Whilst associations between polygenic risk scores (PRSs) for schizophrenia and various phenotypic outcomes have been reported, an understanding of developmental pathways can only be gained by modelling comorbidity across psychopathology. We examine how genetic risk for schizophrenia relates to adolescent psychosis-related and internalizing psychopathology using a latent modelling approach, and compare this to genetic risk for other psychiatric disorders, to gain a more comprehensive understanding of the developmental pathways at this age. PRSs for schizophrenia, major depressive disorder, neuroticism and bipolar disorder were generated for individuals in the Avon Longitudinal Study of Parents and Children (ALSPAC) birth cohort. Multivariate linear regression was used to examine the relationships of these PRSs with psychopathology factors modelled within (i) a correlated factors structure and (ii) a bifactor structure. The schizophrenia PRS was associated with an increase in factors describing psychotic experiences, negative dimension, depression and anxiety, but, when modelling a general psychopathology factor based on these measures, specific effects above this persisted only for the negative dimension. Similar factor relationships were observed for the neuroticism PRS, with a (weak) specific effect only for anxiety once modelling general psychopathology. Psychopathology during adolescence can be described by a general psychopathology construct that captures common variance as well as by specific constructs capturing remaining non-shared variance. Schizophrenia risk genetic variants identified through genome-wide association studies mainly index negative rather than positive symptom psychopathology during adolescence. This has potentially important implications both for research and risk prediction in high-risk samples.

## Introduction

Most psychiatric disorders are of complex multifactorial aetiology^1^, with genome-wide association studies (GWASs) indicating that multiple loci contribute to the aetiology of schizophrenia, bipolar disorder, depression and anxiety disorders, with evidence, provided by family and GWA studies, of partly shared genetic effects^2–12^.

Studying the phenotypic manifestations of genetic liability for psychiatric disorders in the general population can provide an understanding of the developmental pathways and risk prediction. Although individual loci have small effects on risk, cumulatively, alleles on current GWAS platforms explain a substantial proportion of genetic variation^13,14^. Information from even moderately associated alleles can be collapsed into a single polygenic risk score (PRS) that can be used to explore how genetic risk is manifested early during development^15^.

We previously examined the psychopathological features associated with early expression of genetic risk for schizophrenia in a large birth-cohort study, and found strong evidence that a schizophrenia PRS was associated with negative symptoms and anxiety during adolescence, but only very weak evidence of association with psychotic experiences at this age^16^. However, we were not able to tease out comorbidity across disorders, nor deal with measurement error that might explain the weaker evidence of association with psychotic experiences than with negative symptoms or anxiety.

One approach to address these limitations is to use confirmatory factor analysis (CFA) to explore the structure of psychopathology in a latent modelling framework. Such analyses can be used to estimate the co-variance between psychopathologies and effectively model the measurement error present in the data. In the CFA approach, error variance is separated from the shared variance that is thought to be due to the underlying construct. As a consequence, a resultant latent variable is considered to be a more precise depiction of a phenotype than either its manifest variables or a sum-score derived from them^17,18^. One such latent modelling framework used to investigate the common symptom structures of psychological domains is the bifactor model, also known as the general-specific model^19–21^. Bifactor models reflect the notion that variability in a specific item response may be due to multiple underlying sources rather than the true score plus error approach considered in standard latent trait modelling. For example, Caspi et al.^19^ describe a general psychopathology (*p*) factor, analogous to the *g* factor of general intelligence, which captures commonalities between externalizing, internalizing, and thought disorder symptoms. This general psychopathology factor has been suggested to reflect shared elements of psychiatric disorder aetiology, including genetic vulnerability^22,23^.

A systematic review of the phenotypic correlational structure from behavioural genetic studies provides support for a hierarchical structure of first- and higher-order dimensions of psychopathology^24^. Given that genetic effects on psychopathology are likely to consist of both highly pleiotropic and dimension-specific effects, studying such effects will be enhanced through modelling such a hierarchical structure compared to examining specific disorders as outcomes^24^. This approach is consistent with the cross-cutting approach described by the NIMH RDoC initiative towards mental health research^25^, though also posits that gains in knowledge can result from studying higher-order constructs of psychopathology. Understanding heterogeneity can be further enhanced by comparing effects of multiple exposures within such a hierarchical model to understand the different patterns of exposure risk related to dimensions of psychopathology^24^.

We therefore aimed to use a latent modelling framework to: (i) determine which type of latent model, including a bifactor model, best describes the pattern of psychosis-related and internalizing psychopathology during adolescence in the general population, (ii) examine how genetic risk for schizophrenia relates to the latent constructs described within such a model (hence addressing issues of comorbidity and measurement error that limited previous interpretations of our data), and (iii) examine whether the pattern of associations for schizophrenia genetic risk is similar, or different, to that of genetic risk for neuroticism, depression and bipolar disorder.

## Methods and materials

### Participants

The sample comprised of individuals (initially 14,062 children) within the Avon Longitudinal Study of Parents and Children (ALSPAC) birth cohort (www.alspac.bris.ac.uk, see http://www.bris.ac.uk/alspac/researchers/data-access/data-dictionary for all available data)^26,27^. All subjects provided written informed consent, and ethical approval for the study was obtained from the ALSPAC Ethics and Law Committee and the Local Research Ethics Committees.

To maximize sample size, and to limit the influence of age or measurement source, psychopathology measures were assessed using responses to self-report items as close as possible to age 16 years.

### Measures

#### Psychotic experiences

Ten items from the self-report Psychosis-Like Symptoms Questionnaire (PLIKS-Q)^28^ at age 16.5 years, rated on a 3-point scale (never; maybe; definitely), were used to indicate a psychotic experiences latent factor. Items assessed presence of hallucinations, delusions and thought interference since age 15 (see Supplementary Table 1 for more details of all measures).

#### Negative dimension

Eleven items from the negative symptom subscale of the validated^29,30^ Community Assessment of Psychic Experiences (CAPE) self-report questionnaire at age 16.5 years, rated on a 4-point scale (never; sometimes; often; nearly always), were used to indicate a negative dimension latent factor, representing the negative or “loss of function” symptoms associated with psychosis such as apathy, anergia and asociality. Items used measured the symptoms experienced in the past month.

#### Depression

Thirteen self-report items (rated as not true; sometimes true; true) from the Mood and Feelings Questionnaire (MFQ)^31^ measuring past 2-weeks depressive symptoms at age 16.5 years were used to indicate a depression latent factor.

#### Anxiety

Anxiety items were taken from the semi-structured Development and Well Being Assessment (DAWBA) questionnaire at age 15.5 years, a valid instrument in community and clinical samples^32^. Seventeen items related to past-month generalized anxiety disorder and agoraphobia were used to indicate an anxiety latent factor.

#### Polygenic risk scores

Following quality control and imputation, genetic data were available for 8252 unrelated individuals (further details in Supplementary Methods). PRSs for schizophrenia, major depressive disorder (MDD), neuroticism and bipolar disorder were constructed, as described previously^13,16^, using GWAS summary statistics from discovery studies^33–36^ (Table 1).

**Table 1:** Discovery study GWASs and number of SNPs used to generate PRSs for each trait of interest

		Number of SNPs in each PRS			
Trait	Discovery study	*P*_T_ = 0.5	*P*_T_ = 0.05	*P*_T_ = 1e − 5	GWS
Schizophrenia	2014 PGC GWAS^33^	191,361	47,960	737	111
MDD	2016 Hyde et al. GWAS^34^	292,257	53,937	145	8
Neuroticism	2016 Smith et al. GWAS^35^	265,332	49,811	147	9
Bipolar disorder	2011 PGC GWAS^36^	114,262	22,154	34	4

*SNPs* single nucleotide polymorphisms, *PRS* polygenic risk score, *P*_T_ discovery study trait association *P*-value threshold used to include SNPs in PRS, *GWS* independent genome-wide significant SNPs reported by discovery study, *PGC* Psychiatric Genomics Consortium, *GWAS* genome-wide association study, *MDD* major depressive disorder

PRSs were calculated for each ALSPAC individual using PLINK (v1.07)^37^ by summing the number of risk alleles for each single nucleotide polymorphism (SNP) weighted by its discovery sample effect size (further details in Supplementary Methods). Our primary analysis used standardized scores generated from a list of SNPs with a GWAS discovery sample *P*-value threshold (*P*_T_) ≤ 0.05. Correlations between PRSs at *P*_T_ ≤ 0.05 ranged from −0.031 to 0.195 (Supplementary Table 2). As a secondary analysis, PRSs were also generated using SNPs meeting 0.5, 1e^−5^, and genome-wide level *P*-value thresholds. For all discovery studies, genome-wide significance was defined as *P* ≤ 5e^−8^, with the exception of the MDD discovery study^34^ where *P* ≤ 1e^−8^ was considered genome-wide significant due to the 15 million SNPs in the data used within the study from 23andMe, Inc., a personal genetics company.

### Statistical analyses

Statistical analyses were conducted using Mplus (version 7.31)^38^. Individuals were included in the analysis if they had taken part in all psychopathology measures (*N* = 3650). The analysis sample was more likely to be female and came from more advantaged backgrounds (Supplementary Table 3).

**Fig. 1:**
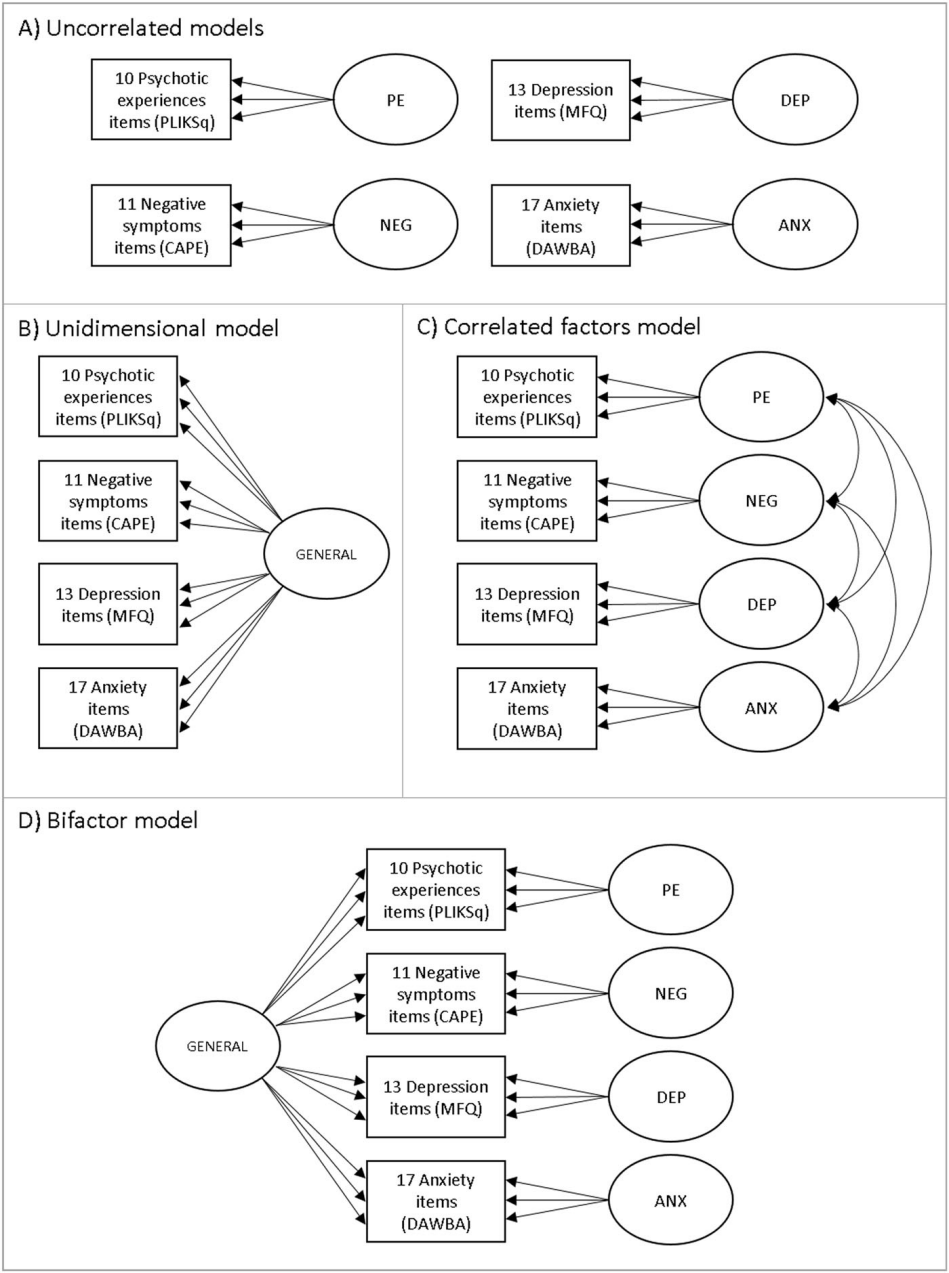
Measurement models developed to explore the dimensional structure of the items and the relationship between psychotic experiences (PE), negative dimension (NEG), depression (DEP), anxiety (ANX) and general psychopathology (GENERAL). Boxes represent multiple individual items relating to each domain. Each of these items would load onto a factor; however, for simplicity, only three arrows are shown emerging from each factor

Ordinal items from each questionnaire (PLIKS-Q, CAPE, MFQ and DAWBA) were used as indicators of latent constructs. To explore the dimensional structure of the items and the relationship between psychotic experiences, negative dimension items, depression and anxiety, four measurement models were estimated and compared (Fig. 1): (i) four uncorrelated latent variables for psychotic experiences, negative dimension, anxiety and depression, (ii) a unidimensional model consisting of a single latent variable corresponding to a common general psychopathological trait, (iii) four correlated latent variables for psychotic experiences, negative dimension, anxiety and depression and (iv) a bifactor model consisting of a single latent variable corresponding to an underlying unidimensional general psychopathological trait and four specific latent variables for psychotic experiences, negative dimension, anxiety and depression.

Mean and variance-adjusted weighted least squares (WLSMV) estimation was used to estimate each model^18^. Absolute model fit was evaluated using the root mean square error of approximation (RMSEA)^39^ index, the comparative fit index (CFI)^40^ and the Tucker–Lewis Index (TLI)^41^. All candidate models were also re-estimated using full information maximum likelihood with robust standard errors (MLR) to obtain Akaike Information Criterion (AIC)^42^, Bayesian Information Criterion (BIC)^43^ and sample size adjusted BIC (ssaBIC) relative fit measures. Due to the number of dimensions and, subsequently, the very large number of integration points needed to estimate the models, we used WLSMV for all subsequent association analyses as it was computationally more efficient when modelling ordinal data. To assess model reliability, omega reliability coefficients^44^ were calculated (see Supplementary Methods for further detail).

Multivariate linear regression (i.e. modelling all outcomes simultaneously) was used to examine the relationships between the standardized PRSs and psychopathological factors in the correlated factors and bifactor models. No covariates accounting for possible population ancestry effects were included as ALSPAC has previously been shown to have no significant population stratification and genome-wide analyses with other phenotypes indicate a low lambda^45–48^. Individuals were included in the association analyses if they had responded to at least four questions per psychopathology measure and had genetic data available (*N* *=* 2863). As a sensitivity analysis to correct for multiple testing, factor scores were exported from Mplus and permutation-adjusted *P*-values for associations with the PRSs were computed using R^49^ (Supplementary Table 4). We also investigated the potential of bias within our analyses due to systematic differences between our analysis sample (2863 individuals with genetic and phenotypic data) and individuals not included in our analyses (5389 individuals with genetic data but no phenotypic data). To do this, we generated a set of weighted results using inverse probability weighting (IPW)^50^ (see Supplementary Methods for further detail).

## Results

### Model fit

Modelling the data within a bifactor structure described the data better than uncorrelated, unidimensional or correlated factor structures, providing the lowest AIC, BIC and ssaBIC values (Table 2). Both the correlated factors and bifactor models showed excellent fit across all absolute fit statistics (Table 2).

**Table 2:** Model fit statistics for the four measurement models (*N* = 3650)

Model	Number of parameters	AIC^a^	BIC^a^	ssaBIC^a^	RMSEA (90% CI)	CFI	TLI
Uncorrelated group factors	146	183486.4	184392.0	183928.0	0.091 (0.090, 0.091)	0.620	0.604
Unidimensional	146	188747.9	189653.5	189189.6	0.060 (0.059, 0.060)	0.835	0.828
Correlated group factors	152	180413.7	181356.5	180873.5	0.029 (0.028, 0.030)	0.961	0.959
Bifactor	197	179668.5	180890.4	180264.4	0.028 (0.027, 0.029)	0.965	0.962

*AIC* Akaike Information Criterion, *BIC* Bayesian Information Criterion, *ssaBIC* sample size adjusted Bayesian Information Criterion, *RMSEA* root mean square error of approximation, *CFI* comparative fit index, *TLI* Tucker–Lewis index

^a^Estimated using Monte Carlo integration with 8000 integration points

Although the model fit statistics indicated that the bifactor model provided the best fit, bifactor models can be difficult to interpret and concerns have been raised that they may over-fit data by capturing unwanted noise as well as construct relevant variance^51^. Therefore, we present results for both the correlated and bifactor models in the aim of adding robustness to our findings and to allow for easier interpretation of the patterns of association between genetic liability for schizophrenia, MDD, neuroticism and bipolar disorder and adolescent psychopathology.

### Item loadings, correlations and reliability

All items had standardized factor loadings >0.4 onto their corresponding latent variables within the correlated factors model (Supplementary Table 1). Correlations between the four latent variables are shown in Supplementary Table 5 and ranged from 0.410 (negative dimension and anxiety) to 0.723 (negative dimension and depression).

All negative dimension and depression item factor loadings were highest for the general factor within the bifactor model. In contrast, almost all psychotic experiences and anxiety item factor loadings were highest for their corresponding specific factors.

Omega reliability coefficients are shown in Supplementary Table 1. The proportions of variance explained by the specific factors once partialling out the general factor were lower than the corresponding *ω*_S_ estimates, especially for the depression factor (*ω*_S_ = 0.96; *ω*_HS_ = 0.15) indicating that the MFQ items contain little specific variance over and above the general factor. However, the difference between the share of the score variance as a result of all factors and the general factor (*ω* = 0.97; *ω*_H_ = 0.79) indicates that a proportion of the score variance was as a result of the four specific factors of the model.

### Associations between PRSs and psychopathology

**Fig. 2:**
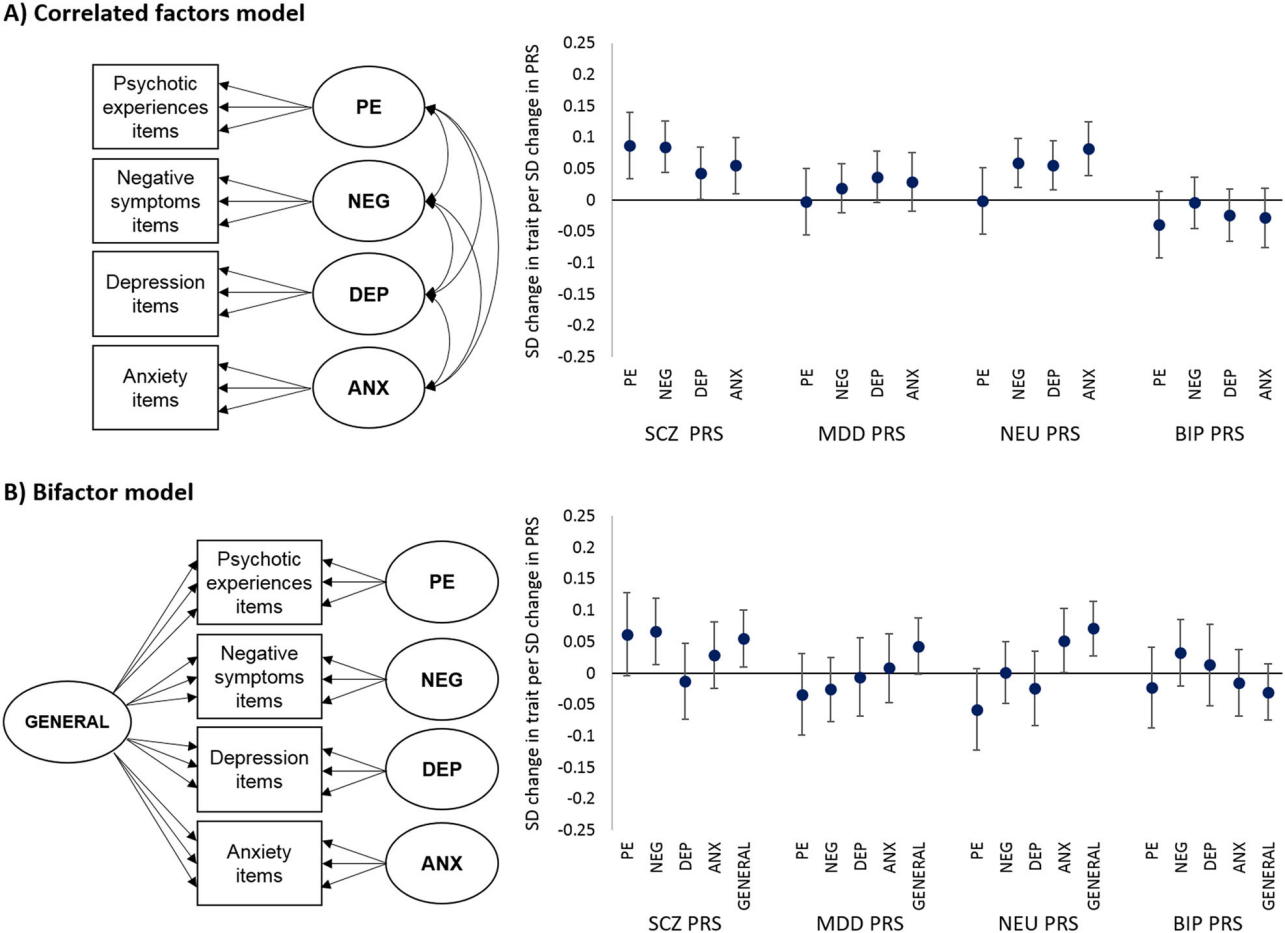
Associations between latent traits for psychotic experiences (PE), negative dimension (NEG), depression (DEP), anxiety (ANX) and general psychopathology (GENERAL) generated using a correlated factors model (a) and a bifactor model (b) and polygenic risk scores (PRS) for schizophrenia (SCZ), major depressive disorder (MDD), neuroticism (NEU) and bipolar disorder (BIP) generated using lists of SNPs meeting a 0.05 *P*-value threshold. Standard deviation (SD) changes in latent trait per SD change in PRS are shown with upper and lower 95% confidence intervals. *N* = 2863

Results for *P*_T_ ≤ 0.05 are shown in Fig. 2 and Table 3. Note that all regression betas (*β*) represent a standard deviation change in factor per standard deviation change in PRS.

**Table 3:** Associations between latent traits, generated using a correlated factors and bifactor model, and polygenic risk scores (PRSs) for psychiatric disorders generated using lists of SNPs meeting a *P*-value threshold of 0.05

		Correlated factors model				Bifactor model			
PRS trait	Outcome	*β* ^a^	LCI	UCI	*P*	*β* ^a^	LCI	UCI	*P*
SCZ	PE	0.087	0.034	0.140	0.001	0.062	−0.005	0.129	0.067
	NEG	0.085	0.044	0.126	<0.001	0.066	0.013	0.119	0.012
	DEP	0.043	0.002	0.084	0.042	−0.013	−0.074	0.048	0.670
	ANX	0.055	0.010	0.100	0.018	0.029	−0.024	0.082	0.287
	GENERAL	–	–	–	–	0.055	0.010	0.100	0.014
MDD	PE	−0.002	−0.055	0.051	0.933	−0.034	−0.099	0.031	0.293
	NEG	0.019	−0.020	0.058	0.347	−0.026	−0.077	0.025	0.310
	DEP	0.037	−0.004	0.078	0.084	-0.006	−0.069	0.057	0.859
	ANX	0.029	−0.018	0.076	0.225	0.008	−0.047	0.063	0.774
	GENERAL	–	–	–	–	0.043	−0.002	0.088	0.059
NEU	PE	−0.001	−0.054	0.052	0.972	−0.058	−0.123	0.007	0.080
	NEG	0.059	0.020	0.098	0.003	0.001	−0.048	0.050	0.959
	DEP	0.055	0.016	0.094	0.007	−0.024	−0.083	0.035	0.420
	ANX	0.082	0.039	0.125	<0.001	0.052	0.001	0.103	0.042
	GENERAL	–	–	–	–	0.071	0.028	0.114	0.001
BIP	PE	−0.039	−0.092	0.014	0.156	−0.023	−0.088	0.042	0.486
	NEG	−0.004	−0.045	0.037	0.841	0.033	−0.020	0.086	0.222
	DEP	−0.024	−0.065	0.017	0.264	0.013	−0.052	0.078	0.684
	ANX	−0.028	−0.075	0.019	0.243	−0.015	−0.068	0.038	0.576
	GENERAL	–	–	–	–	−0.030	−0.075	0.015	0.181

*SCZ* schizophrenia, *MDD* major depressive disorder, *NEU* neuroticism, *BIP* bipolar disorder, *PE* psychotic experience, *NEG* negative dimension, *DEP* depression, *ANX* anxiety, *GENERAL* general psychopathology, *LCI* lower 95% confidence interval, *UCI* upper 95% confidence interval, *P* *P-*value for association between latent trait and PRS

^a^Standardized estimate

#### Schizophrenia PRS

When modelling adolescent psychopathology within a correlated factors model, an increase in the schizophrenia PRS was associated with an increase in all psychopathology factors (psychotic experiences: *β*, 0.09; 95% CI, 0.03–0.14; *P* = 0.001; negative dimension: *β*, 0.09; 95% CI, 0.04–0.13; *P* < 0.001; depression: *β*, 0.04; 95% CI, 0.00–0.08; *P* = 0.042; anxiety: *β*, 0.06; 95% CI, 0.01–0.10; *P* = 0.018). When introducing the general psychopathology factor within the bifactor model, the schizophrenia PRS was associated with an increase in the negative dimension factor (*β*, 0.07; 95% CI, 0.01–0.12; *P* = 0.012) and general psychopathology factor (*β*, 0.06; 95% CI, 0.01–0.10; *P* = 0.014). There was weaker evidence that the schizophrenia PRS was associated with an increase in the psychotic experiences factor (*β*, 0.06; 95% CI, −0.01 to 0.13; *P* = 0.067), whilst no association with depression or anxiety factors was observed.

#### Neuroticism PRS

There was evidence that an increase in the neuroticism PRS was associated with an increase in the negative dimension (*β*, 0.06; 95% CI, 0.02–0.10; *P* = 0.003), depression (*β*, 0.06; 95% CI, 0.02–0.10; *P* = 0.007) and anxiety (*β*, 0.08; 95% CI, 0.04–0.13; *P* < 0.001) latent factors within the correlated factors model. Within the bifactor model, there was strong evidence that neuroticism PRS was associated with an increase in the general psychopathology factor (*β*, 0.07; 95% CI, 0.03–0.11; *P* = 0.001) and, although weaker, with an increase in the anxiety factor (*β*, 0.05; 95% CI, 0.00–0.10; *P* = 0.042). There was weak evidence that the neuroticism PRS was associated with a decrease in the psychotic experiences factor (*β*, −0.06; 95% CI, −0.12 to 0.01; *P* = 0.080).

#### Bipolar disorder and MDD PRSs

The MDD PRS was weakly associated with depression in the correlated factors model (*β*, 0.04; 95% CI, −0.00 to 0.08; *P* = 0.084) and with the general factor in the bifactor model (*β*, 0.04; 95% CI, −0.00 to 0.09; *P* = 0.059). There was no robust evidence of an association between psychopathology factors and the bipolar disorder PRS when modelled within a correlated factors or bifactor model.

#### Sensitivity analyses

Very similar results were observed for PRSs generated using SNPs with a trait association of *P* ≤ 0.5. However, results for PRSs derived using lower (1e^−5^ or genome-wide level of association) *P*-value thresholds were more inconsistent and did not follow any clear patterns across sensitivity *P*-thresholds (Supplementary Figure 1; full results available on request).

Interpretation of associations using permuted *P*-values was substantively the same as above, with exception of the associations between the schizophrenia PRS and psychotic experiences, the neuroticism PRS and psychotic experiences and anxiety, and the MDD PRS and the general factor where the strength of evidence was now considerably weaker (Supplementary Table 4).

A comparison of the IPW results to results without weighting for potential bias due to missingness can be found in Supplementary Tables 6 and 7. Estimates and standard errors of the association between PRSs and psychopathology domains within the correlated and bifactor models were similar between weighted and unweighted analyses although the association between the schizophrenia PRS and negative dimension within the bifactor model was less robust (Supplementary Table 7).

## Discussion

### Correlated factors model

When modelling psychotic experiences, negative dimension items, depression and anxiety as separate, correlated, latent constructs (correlated factors model), we found that genetic risk for schizophrenia was associated with an increase in all four adolescent psychopathology constructs. Schizophrenia PRS association effect sizes were similar and confidence intervals overlapped across all psychopathology factors. The smallest effect size was for the association between the schizophrenia PRS and depression which is consistent with our previous publication using binary outcome measures within this sample^16^. Our results from the correlated factors model showing stronger evidence of association with psychotic experiences compared to our previous publication^16^, suggest that accounting for measurement error through use of latent models might be particularly important for these phenomena.

### Bifactor model

Bifactor models have a number of advantages over standard univariate approaches and are a popular approach in modelling construct-relevant multidimensionality^52,53^, improving psychiatric phenotype definition and, in comparison to a summed-score approach, can provide higher statistical power to detect larger effect sizes^54^. Bifactor models have been used in twin studies to decompose additive genetic and environmental effects across phenotypes^55,56^ and in a cohort study to investigate associations with candidate genes implicated in affective disorders^54^, but have not been utilized previously to understand phenotypic manifestation of polygenic liability for psychiatric disorders as far as we are aware.

The high correlation between the psychopathology factors and large share of the score variance as a result of the general factor (indicated by the omegas) suggest that covariance between responses to items measuring psychotic experiences, negative dimension, depression and anxiety can be explained by an underlying general psychopathology latent construct within the general population, distinct from latent constructs specific for each trait. In comparison to the correlated model results, there was only weak evidence of association between schizophrenia genetic risk and remaining variance for psychotic experiences after accounting for the general psychopathology factor. This suggests that psychotic experiences resulting from higher genetic risk for schizophrenia usually occur, at this age, in the presence of other psychopathology too. This is perhaps not surprising; for example, it is hard to imagine holding paranoid beliefs or hearing hostile voices without some comorbid anxiety or low mood.

The evidence of association between schizophrenia genetic risk and remaining variance for anxiety, and especially for depression, was even weaker when taking into account the general psychopathology factor. However, there was stronger evidence of association with the remaining variance relating to the negative dimension items, although our IPW results suggest that this association might not be robust. This indicates that schizophrenia genetic risk may manifest particularly strongly as negative dimension traits in adolescence, above and beyond the occurrence of general psychopathology, confirming our previous observation^16^. It is also possible that risk variants for schizophrenia identified in the GWAS may only weakly index risk for hallucinations and delusions and more strongly reflect genetic risk for other characteristics such as negative symptoms that index severity or chronicity of illness and that might be selected for in clinically ascertained samples^57^.

### Interpretation in context of previous studies

Whilst family studies have shown that negative symptoms may have higher familial aggregation compared to positive or depressive symptoms in people with schizophrenia^58^, as yet there are no clear patterns of heritability in clinical samples for phenotype dimensions as they are currently conceived^59,60^. A population-based twin study of trait psychopathology showed that self-reported anhedonia and parent-rated negative symptoms were more heritable than hallucinations, though no more heritable than paranoia^61^. Our findings indicate that negative dimension traits as well as other psychopathology during adolescence, whilst not necessarily at levels of clinical significance, are indeed influenced by common genetic variants that increase the risk for schizophrenia.

Other studies have examined the relationship between schizophrenia genetic risk and psychopathology, both in clinical and population-based samples. One study of people with schizophrenia reported that polygenic risk was associated with negative/disorganized factor scores but not with positive symptom or mood dimensions^62^, and more recently associations were reported between genetic risk scores and both anxiety symptoms and general psychopathology, but not with positive or negative symptom dimensions, in patients with first episode psychosis^63^. Our correlated model findings are consistent with associations reported with depression and anxiety in ALSPAC and the Netherlands Twin Register^64^. Other studies have not found evidence of associations between schizophrenia genetic risk and dimensions of psychopathology^65,66^, although statistical power may have been limited due to the size of the discovery or target samples used.

The lack of consistency of findings across studies to date may be partly due to the difficulty of teasing out psychopathology-specific effects from those that are shared across symptom domains. By using a bifactor modelling approach, our study is the first to test whether genetic risk is manifest as a common psychopathology, or as specific symptoms related to one or more underlying psychopathology constructs. Whilst we show that genetic risk for schizophrenia is manifested primarily as general psychopathology and possibly negative dimension traits, it is possible that with greater power, for example from risk scores derived using yet larger discovery samples, we might also find evidence of specific effects on psychotic experiences, anxiety and depression above and beyond the effect on general psychopathology. This might be difficult, however, as specific traits appear to offer very little variability above that explained by general psychopathology at this age. More detailed analyses, for example using risk scores for specific sets of functionally related genes or more detailed psychopathology items, might also allow us to better understand the biological pathways that lead to specific, as well as shared, psychopathology through use of approaches such as latent trait modelling as we use here.

### Genetic risk for MDD, neuroticism and bipolar disorder

We found no robust evidence of association between the bipolar disorder genetic risk score and adolescent psychopathology, though this might be due to the smaller discovery sample used to derive PRSs for this phenotype compared to those for schizophrenia, MDD and neuroticism. As compared to the schizophrenia and neuroticism associations, the MDD PRS was only weakly associated with the general factor which may be due to the lower SNP-based heritability for MDD reported by the GWAS used within the current study (0.06–0.07)^34^ as compared to the other phenotypes.

We found that genetic risk for neuroticism was strongly associated with anxiety, depression and negative dimension constructs within the correlated factors model but, unlike our results for schizophrenia genetic risk, not with psychotic experiences. Within the bifactor model, genetic risk for neuroticism was strongly associated with the general psychopathology construct, and less strongly with the remaining variance for anxiety. Evidence for association with remaining variance for negative dimension items as well as that for psychotic experiences was weaker than those for schizophrenia genetic risk, indicating that genetic risk for schizophrenia may have a more specific effect on these phenotypes than genetic risk for neuroticism.

### Strengths and limitations

The use of a large population-based sample with a broad range of measures of psychopathology during adolescence allows us to infer how genetic risk for psychiatric disorders is likely manifested in the general population at this age. However, whilst the ALSPAC cohort is broadly representative of the UK population, attrition and missing data means that selection bias might have affected our results. Genetic risk for schizophrenia is associated with increased likelihood of attrition^67^, and if presence of psychopathology is also related to missingness this could introduce collider bias in our results.

Whilst self-report measures may perform less well for psychotic experiences than other psychopathological domains, we used self-report measures as we wanted all psychopathology domains assessed at similar ages using questionnaire data, and we previously reported that associations with schizophrenia genetic risk were consistent when comparing self-report and interview-assessed psychotic experiences^16^. Unfortunately, additional data were not collected to ascertain the test–retest reliability of the questionnaires used. We can therefore not assess whether intra-individual variability in responses has biased our results.

A strength of our study is that use of a latent modelling framework allowed us to tease out the effects that explain the shared variance across measures from those that are specific to constructs separate from general psychopathology. However, we do not know the source or relevance of the specific-construct variance, particularly where this has only modest specific variance over and above the general factor, as for example, the negative dimension construct. Furthermore, whilst the symptoms assessed using the CAPE measure of negative symptoms were derived from the Scale for Assessment of Negative Symptoms and load onto a separate factor from depressive symptoms in other studies^29^, as in ours, they might not accurately index negative symptoms as conceptualized in schizophrenia.

Furthermore, item contamination may have occurred, whereby, for example, similarity in items between the 12-item Eysenck Personality Questionnaire-Revised used to generate the neuroticism PRS and the depression and anxiety measures used in our study may have led to an overestimate of association between the neuroticism PRS and the general factor. However, a previous study identified a genetic overlap between neuroticism (negative emotionality) and general psychopathology using an item pool designed to exclude synonyms or antonyms of psychopathology symptoms^56^, suggesting that such a bias is unlikely to adequately explain our findings. Similarity in question wording was also evident between the CAPE and MFQ items used to construct the negative dimension of psychosis and depression factors, respectively. For example, both scales contain items relating to loss of motivation. This may explain their high correlation within the correlated factors model.

The particular measures used in the current study may also have introduced confounding by question time-frames. Questions from two domains referred to past month experiences, one to experiences in the past 2 weeks and one to experiences since age 15 years. This may reduce/increase the covariances between each pair of latent factors and hence the degree of support for the bifactor model.

Finally, our models do not include measures of externalizing psychopathology or cognition, thus limiting comparison to the general factor in bifactor models that have been derived in studies that have incorporated such measures.

## Conclusions

Psychopathologies experienced during adolescence share common variance that may be captured by a general psychopathology construct with remaining, non-shared, variance representing what is distinct to each specific symptom domain. Genetic risk for schizophrenia is manifested primarily as general psychopathology encompassing a mixture of psychotic, negative dimension, anxiety and depressive symptoms, along with potentially specific effects on negative dimension items. GWAS of symptom dimensions utilizing a latent modelling framework might be able to add to our understanding of biological pathways that influence specific phenotypes to a greater extent than GWAS of schizophrenia per se, if power issues from the limited sample sizes with rich phenotypic data could be overcome.

## Electronic supplementary material

Supplementary material
