# Supplementary material for: Investigating the genetic architecture of general and specific psychopathology in adolescence
Source: Transl Psychiatry. 2018 Aug 8;8:145. doi: 10.1038/s41398-018-0204-9 (PMC6082910; doi:10.1038/s41398-018-0204-9)
Supplement: Supplementary file 1 — Supplementary material [file 41398_2018_204_MOESM1_ESM.doc]

Investigating the genetic architecture of general and specific psychopathology in adolescence -- Supplementary Material

Supplementary Methods

Genetic data

Genetic data were acquired using the Illumina HumanHap550 quad genome-wide single nucleotide polymorphism (SNP) genotyping platform from 9912 participants. Individuals were excluded from further analysis based on gender mismatches, minimal or excessive heterozygosity, disproportionate levels of individual missingness (>3%), evidence of cryptic relatedness (>10% of alleles identical by descent), and being of non-European ancestry (assessed by multidimensional scaling analysis including HapMap 2 individuals). SNPs with a minor allele frequency (MAF) of < 1%, Impute2 information quality metric of < 0.8, a call rate of < 95% or evidence for violations of Hardy-Weinberg equilibrium (P-value < 5e-7) were removed. Imputation of the target data was performed using Impute V2.2.2 against the 1000 genomes reference panel (Phase 1, Version 3; all polymorphic SNPs excluding singletons), using 2186 reference haplotypes (including non-Europeans). Following quality control assessment and imputation and restricting to 1 young person per family, genetic data was available for 8252 ALSPAC individuals.

Polygenic risk scores

Polygenic risk scores (PRSs) for schizophrenia, major depressive disorder (MDD), neuroticism and bipolar disorder were constructed, as described previously,^1, 2^ using GWAS summary statistics from discovery studies.^3-6^

SNPs were excluded from the analysis if they had a minor allele frequency less than 0.01, imputation quality less than 0.8, or if there was allelic mismatch between samples (alleles reported by the discovery study not matching alleles in the ALSPAC sample). Due to the high linkage disequilibrium (LD) within the extended major histocompatibility complex (MHC; chromosome 6: 25-34Mb) only a single SNP was included to represent this region. Remaining SNPs were then further pruned for LD using the PLINK (v1.90)^7^ ‘clump’ command to retain SNPs with a trait association *P*-value ≤ 0.5 and *r*^2^ < 0.25 within 500kb windows.

Polygenic scores were calculated for each ALSPAC individual using PLINK (v1.07)^7^ by summing the number of risk alleles for each SNP (0, 1 or 2) weighted by the logarithm of its discovery sample odds ratio (OR) for binary traits (schizophrenia, MDD, bipolar disorder) or by the beta for continuous traits (neuroticism). Our primary analysis used scores generated from a list of SNPs with a GWAS training-set *P*-value threshold (*P*_T_) ≤ 0.05. As a secondary analysis, PRSs were also generated using SNPs meeting 0.5, 1e-5, and genome-wide level *P*-value thresholds. For all discovery studies, genome-wide significant was defined as *P*≤5e-8, with the exception of the MDD discovery study^4^ where *P*≤1e-8 was considered genome-wide significant due to the 15 million SNPs in the data used within the study from 23andMe, Inc., a personal genetics company.

Model fit

Absolute model fit was evaluated using the Root Mean Square Error of Approximation (RMSEA)^8^ index, the comparative fit index (CFI)^9^ and the Tucker-Lewis Index (TLI).^10^ Reasonably good fit between the target model and observed data is considered when RMSEA values are close to or below 0.06, and CFI and TFI values are close to or greater than 0.95.^11, 12^

All candidate models were also re-estimated using full information maximum likelihood (FIML) to obtain Akaike Information Criterion (AIC),^13^ Bayesian Information Criterion (BIC)^14^ and sample size adjusted Bayesian Information Criterion (ssaBIC) measures. These measures allow for direct comparison of plausibility of each four candidate models with lower values indicating better model fit.

Model reliability

For the bifactor model, omega reliability coefficients were calculated to assess the share of the score variance as a result of all factors (omega; ω), the specific factors (omega subscale; ω_S_), the general factor (omega hierarchical; ω_H_) and the specific factors when the general factor is partialled out (omega hierarchical subscale; ω_HS_).^15^

Inverse probability weighting

Inverse probability weighting (IPW) was used to investigate the potential of bias within our analyses due to systematic differences between our analysis sample (2863 individuals with genetic and phenotypic data) and individuals not included in our analyses (5389 individuals with genetic data but no phenotypic data).

We firstly generated a missingness model using potential predictors of whether an individual was in the final analysis or not (missingness). These predictors were strongly associated with missingness (OR>1.2) and had minimal missing data (>7000 data points). Modal imputation was used to replace missing data within each predictor and a multivariable logistic regression was used to indicate which imputed predictors showed the strongest evidence of an association with missingness (*P* < 0.1). The final predictors used were maternal early parenthood/pregnancy, maternal partner status during pregnancy, parental lack of educational qualifications during pregnancy, maternal social network practical support during pregnancy, maternal parity, maternal financial difficulties during pregnancy, sex, whether the individual was part of the ALSPAC Phase I core sample,^16^ maternal partner affection during pregnancy, and maternal psychopathology during pregnancy. The genetic risk scores for schizophrenia, MDD, neuroticism and bipolar disorder were also included in the missingness model.

As the effect of an observed predictor on missingness may depend on which other predictors are observed,^17^ interaction terms were created between each predictor and a missingness flag deemed representative of the amount of missing data within each moderately incomplete predictor. This missingness flag was based on the missingness pattern of the “parental lack of educational qualifications during pregnancy” variable. Ordinary interactions between pairs of predictors were also generated. Forward stepwise selection was used, with threshold *P* < 0.1, to select which of the interaction terms to add to the existing missingness model and both the Hosmer-Lemeshow test^18^ (*P* = 0.93) and Hinkley’s test^19, 20^ (*P* = 0.56) indicated that the final model fit the data well.

Weights were generated by inverting the predicted probabilities estimated by the missingness model. Weights of the 2863 individuals within the final analysis models ranged from 1.27 to 58.22. To assess the effect of large weights on the analysis, additional weight sets were generated after truncation at the 50^th^ percentile (2.48), 75^th^ percentile (3.02), 90^th^ percentile (3.95), 95^th^ percentile (5.33) and 99^th^ percentile (8.94).

Each weight set was used to perform IPW analyses between psychopathology factors within the correlated factor and bifactor models and psychiatric disorder PRSs. IPW results are shown in Supplementary Tables 6 and 7.

References

1. Jones HJ, Stergiakouli E, Tansey KE, Hubbard L, Heron J, Cannon M *et al.* Phenotypic manifestation of genetic risk for schizophrenia during adolescence in the general population. *JAMA Psychiatry* 2016; **73**(3)**:** 221-228.

2. Purcell SM, Wray NR, Stone JL, Visscher PM, O'Donovan MC, Sullivan PF *et al.* Common polygenic variation contributes to risk of schizophrenia and bipolar disorder. *Nature* 2009; **460:** 748-752.

3. Schizophrenia Working Group of the Psychiatric Genomics Consortium. Biological insights from 108 schizophrenia-associated genetic loci. *Nature* 2014; **511:** 421-427.

4. Hyde CL, Nagle MW, Tian C, Chen X, Paciga SA, Wendland JR *et al.* Identification of 15 genetic loci associated with risk of major depression in individuals of European descent. *Nature Genetics* 2016; **48**(9)**:** 1031-1036.

5. Smith DJ, Escott-Price V, Davies G, Bailey ME, Colodro-Conde L, Ward J *et al.* Genome-wide analysis of over 106 000 individuals identifies 9 neuroticism-associated loci. *Molecular Psychiatry* 2016; **21**(6)**:** 749-757.

6. Bipolar Disorder Working Group of the Psychiatric Genomics Consortium. Large-scale genome-wide association analysis of bipolar disorder identifies a new susceptibility locus near ODZ4. *Nature Genetics* 2011; **43**(10)**:** 977-983.

7. Purcell S, Neale B, Todd-Brown K, Thomas L, Ferreira MA, Bender D *et al.* PLINK: a tool set for whole-genome association and population-based linkage analyses. *American Journal of Human Genetics* 2007; **81:** 559-575.

8. Steiger JH, Lind JC. Statistically based tests for the number of common factors. *Paper presented at the Annual Spring Meeting of the Psychometric Society*: Iowa City, IA., 1980.

9. Bentler PM. Comparative fit indexes in structural models. *Psychological Bulletin* 1990; **107**(2)**:** 238-246.

10. Tucker LR, Lewis C. A reliability coefficient for maximum likelihood factor analysis. *Psychometrika* 1973; **38**(1)**:** 1-10.

11. Hu LT, Bentler PM. Cutoff criteria for fit indexes in covariance structure analysis: conventional criteria versus new alternatives. *Structural Equation Modeling* 1999; **6**(1)**:** 1-55.

12. Brown TA. *Confirmatory Factor Analysis for Applied Research*. 2nd edn. The Guilford Press: New York, NY, 2015.

13. Akaike H. Information theory and an extension of the maximum likelihood principle. *Selected Papers of Hirotugu Akaike*. Springer1998, pp 199-213.

14. Schwarz GE. Estimating the dimension of a model. *The Annals of Statistics* 1978; **6**(2)**:** 461-464.

15. Rodriguez A, Reise SP, Haviland MG. Applying bifactor statistical indices in the evaluation of psychological measures. *Journal of Personality Assessment* 2016; **98**(3)**:** 223-237.

16. Boyd A, Golding J, Macleod J, Lawlor DA, Fraser A, Henderson J *et al.* Cohort Profile: the 'children of the 90s' - the index offspring of the Avon Longitudinal Study of Parents and Children. *International Journal of Epidemiology* 2013; **42:** 111-127.

17. Seaman SR, White IR. Review of inverse probability weighting for dealing with missing data. *Statistical Methods in Medical Research* 2011; **22**(3)**:** 278-295.

18. Hosmer DW, Lemeshow S. *Applied Logistic Regression*. Wiley: New York, 1989.

19. Kang JDY, Schafer JL. Demystifying double robustness: a comparison of alternative strategies for estimating a population mean from incomplete data. *Stat Sci* 2007; **22**(4)**:** 523-539.

20. Hinkley D. Transformation diagnostics for linear models. *Biometrika* 1985; **72**(3)**:** 487-496.

Supplementary Table 1. Standardized factor loadings for the correlated factors and bifactor measurement models tested (n = 3650)

|  |  | Correlated | | | | Bifactor | | | | |
| --- | --- | --- | --- | --- | --- | --- | --- | --- | --- | --- |
| Item | Item question | PE | NEG | DEP | ANX | PE | NEG | DEP | ANX | GEN |
| PE1 | Young person believes someone else has read their thoughts | 0.600** |  |  |  | 0.545** |  |  |  | 0.357** |
| PE2 | Young person believes they have received special messages via TV/radio or program | 0.703** |  |  |  | 0.600** |  |  |  | 0.422** |
| PE3 | Young person believes they have been followed or spied on | 0.748** |  |  |  | 0.490** |  |  |  | 0.481** |
| PE4 | Young person has heard voices other people couldn't hear | 0.748** |  |  |  | 0.607** |  |  |  | 0.461** |
| PE5 | Young person has ever felt they were under the control of a special power | 0.641** |  |  |  | 0.626** |  |  |  | 0.361** |
| PE6 | Young person has ever seen something/someone other people could not see | 0.738** |  |  |  | 0.575** |  |  |  | 0.461** |
| PE7 | Young person has ever felt their thoughts were being taken out of their head against their will | 0.784** |  |  |  | 0.568** |  |  |  | 0.508** |
| PE8 | Young person has ever felt someone else's thoughts were inserted into their head against their will | 0.770** |  |  |  | 0.534** |  |  |  | 0.502** |
| PE9 | Young person has ever felt their thoughts were so loud people could hear what they were thinking | 0.736** |  |  |  | 0.479** |  |  |  | 0.486** |
| PE10 | Young person has ever felt they were someone really special or had special powers | 0.635** |  |  |  | 0.602** |  |  |  | 0.367** |
| NEG1 | Young person has felt lacking in energy |  | 0.675** |  |  |  | 0.244** |  |  | 0.596** |
| NEG2 | Young person has felt like they are not much of a talker |  | 0.561** |  |  |  | 0.414** |  |  | 0.420** |
| NEG3 | Young person has felt they experience few or no emotions at important events |  | 0.637** |  |  |  | 0.328** |  |  | 0.527** |
| NEG4 | Young person has felt lacking in motivation |  | 0.790** |  |  |  | 0.543** |  |  | 0.601** |
| NEG5 | Young person has felt that they are spending their days doing nothing |  | 0.760** |  |  |  | 0.530** |  |  | 0.575** |
| NEG6 | Young person has felt lacking in get up and go |  | 0.835** |  |  |  | 0.605** |  |  | 0.624** |
| NEG7 | Young person has felt they only have a few hobbies or interests |  | 0.678** |  |  |  | 0.385** |  |  | 0.548** |
| NEG8 | Young person has felt they have no interest in being with other people |  | 0.746** |  |  |  | 0.423** |  |  | 0.604** |

Supplementary Table 1 continued.

| NEG9 | Young person has felt they are not a very lively person | |  | 0.736** |  |  |  | 0.510** |  |  | 0.562** |
| --- | --- | --- | --- | --- | --- | --- | --- | --- | --- | --- | --- |
| NEG10 | Young person has felt they are neglecting their appearance/personal hygiene | |  | 0.614** |  |  |  | 0.289** |  |  | 0.518** |
| NEG11 | Young person has felt they can never get things done | |  | 0.793** |  |  |  | 0.355** |  |  | 0.677** |
| DEP1 | Young person has felt unhappy/miserable | |  |  | 0.783** |  |  |  | 0.377** |  | 0.698** |
| DEP2 | Young person hasn't enjoyed anything at all | |  |  | 0.751** |  |  |  | 0.197** |  | 0.718** |
| DEP3 | Young person has felt so tired they sat around and did nothing | |  |  | 0.609** |  |  |  | -0.135** |  | 0.653** |
| DEP4 | Young person has felt very restless | |  |  | 0.577** |  |  |  | 0.020 |  | 0.582** |
| DEP5 | Young person has felt they were no good anymore | |  |  | 0.907** |  |  |  | 0.444** |  | 0.797** |
| DEP6 | Young person has cried a lot | |  |  | 0.690** |  |  |  | 0.433** |  | 0.583** |
| DEP7 | Young person has found it hard to think properly/concentrate | |  |  | 0.675** |  |  |  | -0.100 |  | 0.715** |
| DEP8 | Young person has hated themselves | |  |  | 0.873** |  |  |  | 0.475** |  | 0.751** |
| DEP9 | Young person has felt they were a bad person | |  |  | 0.816** |  |  |  | 0.444** |  | 0.704** |
| DEP10 | Young person has felt lonely | |  |  | 0.824** |  |  |  | 0.347** |  | 0.747** |
| DEP11 | Young person has thought nobody really loved them | |  |  | 0.845** |  |  |  | 0.520** |  | 0.707** |
| DEP12 | Young person has thought they could never be as good as other kids | |  |  | 0.840** |  |  |  | 0.410** |  | 0.741** |
| DEP13 | Young person has felt they did everything wrong | |  |  | 0.880** |  |  |  | 0.461** |  | 0.764** |
| ANX1 | Compared to others of their age, amount young person has worried about own past behaviour | |  |  |  | 0.671** |  |  |  | 0.555** | 0.359** |
| ANX2 | Compared to others of their age, amount young person has worried about school work/exams | |  |  |  | 0.499** |  |  |  | 0.429** | 0.260** |
| ANX3 | Compared to others of their age, amount young person has worried about burglaries/muggings/bombs | |  |  |  | 0.569** |  |  |  | 0.566** | 0.254** |
| ANX4 | Compared to others of their age, amount young person has worried about own health | |  |  |  | 0.642** |  |  |  | 0.608** | 0.305** |
| ANX5 | Compared to others of their age, amount young person has worried about bad things happening to others | |  |  |  | 0.626** |  |  |  | 0.597** | 0.295** |
| ANX6 | | Compared to others of their age, amount young person has worried about the future – new school/house/job |  |  |  | 0.641** |  |  |  | 0.549** | 0.334** |

Supplementary Table 1 continued.

| ANX7 | Compared to others of their age, amount young person has worried about making and keeping friends |  |  |  | 0.683** |  |  |  | 0.573** | 0.364** |
| --- | --- | --- | --- | --- | --- | --- | --- | --- | --- | --- |
| ANX8 | Compared to others of their age, amount young person has worried about death and dying |  |  |  | 0.647** |  |  |  | 0.621** | 0.300** |
| ANX9 | Compared to others of their age, amount young person has worried about being bullied or teased |  |  |  | 0.654** |  |  |  | 0.648** | 0.290** |
| ANX10 | Compared to others of their age, amount young person has worried about own appearance or weight |  |  |  | 0.704** |  |  |  | 0.562** | 0.388** |
| ANX11 | Young person has had panic attack for no reason |  |  |  | 0.549** |  |  |  | 0.329** | 0.355** |
| ANX12 | Young person has felt afraid of or tried to avoid crowds |  |  |  | 0.725** |  |  |  | 0.502** | 0.445** |
| ANX13 | Young person has felt afraid of or tried to avoid public places |  |  |  | 0.774** |  |  |  | 0.630** | 0.429** |
| ANX14 | Young person has felt afraid of or tried to avoid travelling alone |  |  |  | 0.668** |  |  |  | 0.509** | 0.380** |
| ANX15 | Young person has felt afraid of or tried to avoid being far from home |  |  |  | 0.548** |  |  |  | 0.497** | 0.271** |
| ANX16^1^ | Young person fears/avoids situations that involve a lot of people or meeting new people AND young person avoids social situations because of their fear |  |  |  | 0.547** |  |  |  | 0.429** | 0.305** |
| ANX17^1^ | Young person is worried in general AND young person has worried so much it has upset or interfered with their life |  |  |  | 0.746** |  |  |  | 0.595** | 0.411** |
|  |  |  |  |  | ω | - | - | - | - | 0.970 |
|  |  |  |  |  | ω_S_ | 0.913 | 0.919 | 0.955 | 0.922 | - |
|  |  |  |  |  | ω_H_ | - | - | - | - | 0.786 |
|  |  |  |  |  | ω_HS_ | 0.566 | 0.325 | 0.146 | 0.664 | - |

Note: Development and Well Being Assessment (DAWBA) items related to specific phobias and post-traumatic stress disorder were not included in the analysis due to low correlations with other anxiety items. PE, psychotic experience; NEG, negative dimension; DEP, depression; ANX, anxiety; GEN, general psychopathology; ω, omega; ω_S_, omega subscale; ω_H_, omega hierarchical; ω_HS_, omega hierarchical subscale.

^1^ Gated item pairs, where answering “yes” to an initial question lead onto an additional question, collapsed into single items, ** *P*<0.0001

*Supplementary Table 2. Correlations between polygenic risk scores for psychiatric disorders generated using lists of SNPs meeting P-value threshold of 0.05 (n = 2863)*

|  | SCZ | MDD | NEU |
| --- | --- | --- | --- |
| MDD | 0.110 |  |  |
| NEU | -0.031 | 0.087 |  |
| BIP | 0.195 | 0.043 | 0.050 |

Note: SCZ, schizophrenia; MDD, major depressive disorder; NEU, neuroticism; BIP, bipolar disorder

Supplementary Table 3. Sociodemographic differences between individuals who had taken part in all psychopathology measures and the remainder of the ALSPAC sample

|  |  |  | Taken part in all psychopathology measures? | |  |
| --- | --- | --- | --- | --- | --- |
| Measure |  |  | No | Yes | *P* |
| Sex | male | N (%) | 6092 (54.36) | 1543 (42.31) | <0.001 |
|  | female | N (%) | 5115 (45.64) | 2104 (57.69) |  |
| Highest parental  social class | professional/managerial | N (%) | 4131 (50.48) | 2197 (66.52) | <0.001 |
|  | non-professional/managerial | N (%) | 4052 (49.52) | 1106 (33.48) |  |
| Household income  (quintiles;  lowest income  = 1,  highest income = 5) | 1 | N (%) | 1597 (23.75) | 392 (12.23) | <0.001 |
|  | 2 | N (%) | 1417 (21.08) | 551 (17.19) |  |
|  | 3 | N (%) | 1345 (20.01) | 631 (19.69) |  |
|  | 4 | N (%) | 1225 (18.22) | 761 (23.74) |  |
|  | 5 | N (%) | 1139 (16.94) | 870 (27.15) |  |
| Crowding index | ≤ 1 individual per room | N (%) | 9069 (91.66) | 3270 (97.12) | <0.001 |
|  | > 1 individual per room | N (%) | 825 (8.34) | 97 (2.88) |  |
| Parity | 0 | N (%) | 4157 (42.76) | 1716 (50.44) | <0.001 |
|  | 1 | N (%) | 3413 (35.11) | 1175 (34.54) |  |
|  | 2 | N (%) | 1489 (15.32) | 392 (11.52) |  |
|  | 3+ | N (%) | 663 (6.82) | 119 (3.5) |  |
| Highest maternal  education qualification | CSE | N (%) | 2204 (24.29) | 323 (9.45) | <0.001 |
|  | Vocational | N (%) | 1008 (11.11) | 221 (6.46) |  |
|  | O level | N (%) | 3185 (35.1) | 1142 (33.4) |  |
|  | A level | N (%) | 1796 (19.79) | 1005 (29.39) |  |
|  | Degree | N (%) | 881 (9.71) | 728 (21.29) |  |
| Ethnicity | non-white | N (%) | 263 (2.92) | 63 (1.85) | 0.001 |
|  | white | N (%) | 8729 (97.08) | 3346 (98.15) |  |
| Birthweight (g) | | N | 10 459 | 3436 | <0.001 |
|  |  | Mean | 3371.57 | 3411.14 |  |
|  |  | SD | 594.26 | 537.06 |  |

Note: CSE, Certificate of Secondary Education; SD, standard deviation; *P*, *P*-value for chi-squared test (categorical measures) or t-test (birthweight) of differences between individuals who had taken part in all psychopathology measures and the remainder of the ALSPAC sample

Supplementary Table 4. Permutation adjusted associations between exported latent trait factor scores, estimated using a correlated factors and bifactor model, and polygenic risk scores (PRSs) for psychiatric disorders generated using lists of SNPs meeting *P*-value threshold of 0.05

|  |  | Correlated factors model | | Bifactor model | |
| --- | --- | --- | --- | --- | --- |
| PRS trait | Outcome | Beta^1^ | *P* ^2^ | Beta^1^ | *P* ^2^ |
| SCZ | PE | 0.058 | 0.0006 | 0.021 | 0.7515 |
|  | NEG | 0.071 | 0.0006 | 0.040 | 0.0848 |
|  | DEP | 0.046 | 0.0895 | -0.011 | 0.9997 |
|  | ANX | 0.051 | 0.0132 | 0.021 | 0.9423 |
|  | GENERAL | - | - | 0.058 | 0.0124 |
| MDD | PE | 0.018 | 0.8875 | -0.004 | 1.0000 |
|  | NEG | 0.020 | 0.9429 | -0.013 | 0.9997 |
|  | DEP | 0.036 | 0.3413 | 0.011 | 0.9997 |
|  | ANX | 0.033 | 0.2902 | 0.018 | 0.9860 |
|  | GENERAL | - | - | 0.033 | 0.5840 |
| NEU | PE | 0.030 | 0.2894 | -0.005 | 1.0000 |
|  | NEG | 0.046 | 0.0718 | 0.005 | 1.0000 |
|  | DEP | 0.046 | 0.0667 | -0.005 | 1.0000 |
|  | ANX | 0.053 | 0.0049 | 0.031 | 0.3693 |
|  | GENERAL | - | - | 0.053 | 0.0230 |
| BIP | PE | -0.031 | 0.2852 | -0.022 | 0.7118 |
|  | NEG | -0.006 | 1.0000 | 0.020 | 0.9605 |
|  | DEP | -0.018 | 0.9733 | 0.005 | 1.0000 |
|  | ANX | -0.025 | 0.7122 | -0.014 | 0.9996 |
|  | GENERAL | - | - | -0.022 | 0.9825 |

Note: SCZ, schizophrenia; MDD, major depressive disorder; NEU, neuroticism; BIP, bipolar disorder; PE, psychotic experiences; NEG, negative dimension; DEP, depression; ANX, anxiety; GENERAL, general psychopathology; LCI, lower 95% confidence interval; UCI, upper 95% confidence interval; *P*, *P*-value for association between latent trait factor scores and PRS.

^1^ Note that the estimates will differ from the latent factor estimates as the relationship between the factor scores may differ slightly from the latent factors. The estimated factor scores better approximate true factors the more highly correlated the two are.

^2^ Empirical adjusted *P*-value calculated from 10 000 permutations to correct for multiple testing.

Supplementary Table 5. Correlations between 4 specific psychopathology factors as measured within the correlated factors model (n = 3650)

|  | PE | NEG | DEP |
| --- | --- | --- | --- |
| NEG | 0.505 |  |  |
| DEP | 0.523 | 0.723 |  |
| ANX | 0.456 | 0.410 | 0.461 |

Note: PE, psychotic experience; NEG, negative dimension; DEP, depression; ANX, anxiety

*Supplementary Table 6. Standardised effect sizes and standard errors from associations between latent traits, generated using a correlated factors model, and polygenic risk scores (PRSs) for psychiatric disorders following inverse probability weighting*

|  |  |  | Inverse Probability Weighted (% truncated) | | | | | |
| --- | --- | --- | --- | --- | --- | --- | --- | --- |
|  |  | Unweighted | 50% | 75% | 90% | 95% | 99% | Untruncated |
| PRS trait | Outcome | Beta^1^ (SE) | Beta^1^ (SE) | | | | | |
| SCZ | PE | 0.087 (0.027) | 0.090 (0.028) | 0.089 (0.028) | 0.093 (0.029) | 0.098 (0.029) | 0.102 (0.03) | 0.099 (0.031) |
|  | NEG | 0.085 (0.021) | 0.089 (0.021) | 0.083 (0.021) | 0.078 (0.022) | 0.075 (0.022) | 0.075 (0.024) | 0.064 (0.025) |
|  | DEP | 0.043 (0.021) | 0.049 (0.021) | 0.042 (0.021) | 0.040 (0.022) | 0.043 (0.023) | 0.048 (0.024) | 0.045 (0.024) |
|  | ANX | 0.055 (0.023) | 0.065 (0.023) | 0.064 (0.023) | 0.067 (0.024) | 0.072 (0.024) | 0.071 (0.025) | 0.071 (0.025) |
| MDD | PE | -0.002 (0.027) | -0.001 (0.027) | -0.001 (0.027) | -0.004 (0.028) | -0.007 (0.029) | -0.006 (0.030) | -0.005 (0.030) |
|  | NEG | 0.019 (0.020) | 0.020 (0.020) | 0.019 (0.021) | 0.019 (0.021) | 0.020 (0.022) | 0.025 (0.023) | 0.025 (0.024) |
|  | DEP | 0.037 (0.021) | 0.040 (0.021) | 0.040 (0.022) | 0.040 (0.022) | 0.041 (0.023) | 0.041 (0.024) | 0.042 (0.024) |
|  | ANX | 0.029 (0.024) | 0.035 (0.024) | 0.036 (0.025) | 0.036 (0.025) | 0.038 (0.026) | 0.046 (0.027) | 0.048 (0.027) |
| NEU | PE | -0.001 (0.027) | 0.006 (0.027) | 0.006 (0.027) | 0.005 (0.027) | 0.005 (0.028) | 0.002 (0.028) | 0.008 (0.029) |
|  | NEG | 0.059 (0.020) | 0.061 (0.020) | 0.058 (0.020) | 0.059 (0.021) | 0.062 (0.021) | 0.067 (0.023) | 0.073 (0.025) |
|  | DEP | 0.055 (0.020) | 0.060 (0.020) | 0.059 (0.021) | 0.061 (0.021) | 0.064 (0.021) | 0.067 (0.022) | 0.072 (0.023) |
|  | ANX | 0.082 (0.022) | 0.086 (0.022) | 0.084 (0.022) | 0.084 (0.023) | 0.087 (0.023) | 0.095 (0.024) | 0.101 (0.024) |
| BIP | PE | -0.039 (0.027) | -0.044 (0.027) | -0.045 (0.027) | -0.045 (0.028) | -0.045 (0.028) | -0.043 (0.028) | -0.036 (0.029) |
|  | NEG | -0.004 (0.021) | -0.004 (0.021) | -0.001 (0.021) | 0.005 (0.022) | 0.005 (0.023) | 0.004 (0.024) | 0.005 (0.025) |
|  | DEP | -0.024 (0.021) | -0.024 (0.022) | -0.022 (0.022) | -0.017 (0.022) | -0.018 (0.023) | -0.019 (0.023) | -0.018 (0.024) |
|  | ANX | -0.028 (0.024) | -0.032 (0.024) | -0.032 (0.024) | -0.030 (0.025) | -0.032 (0.025) | -0.035 (0.026) | -0.033 (0.026) |

Note: SCZ, schizophrenia; MDD, major depressive disorder; NEU, neuroticism; BIP, bipolar disorder; PE, psychotic experience; NEG, negative dimension; DEP, depression; ANX, anxiety; SE, standard error. Inverse probability weighted refers to analyses including sampling weights for non-response, where the “% truncated” indicates whether the weights were truncated based on the 50^th^ percentile (2.48), 75^th^ percentile (3.02), 90^th^ percentile (3.95), 95^th^ percentile (5.33) and 99^th^ percentile (8.94).

^1^ Standardized estimate

*Supplementary Table 7. Effect sizes and standard errors from associations between latent traits, generated using a bifactor model, and polygenic risk scores (PRSs) for psychiatric disorders following inverse probability weighting*

|  |  |  | Inverse Probability Weighted (% truncated) | | | | | |
| --- | --- | --- | --- | --- | --- | --- | --- | --- |
|  |  | Unweighted | 50% | 75% | 90% | 95% | 99% | Untruncated |
| PRS trait | Outcome | Beta (SE) | Beta (SE) | | | | | |
| SCZ | PE | 0.062 (0.034) | 0.060 (0.034) | 0.063 (0.035) | 0.070 (0.036) | 0.074 (0.036) | 0.074 (0.036) | 0.072 (0.038) |
|  | NEG | 0.066 (0.027) | 0.065 (0.026) | 0.062 (0.026) | 0.055 (0.027) | 0.048 (0.027) | 0.041 (0.028) | 0.022 (0.031) |
|  | DEP | -0.013 (0.031) | -0.013 (0.032) | -0.022 (0.032) | -0.027 (0.032) | -0.028 (0.032) | -0.033 (0.033) | -0.045 (0.034) |
|  | ANX | 0.029 (0.027) | 0.037 (0.027) | 0.039 (0.027) | 0.044 (0.027) | 0.047 (0.028) | 0.043 (0.029) | 0.042 (0.029) |
|  | GENERAL | 0.055 (0.023) | 0.061 (0.022) | 0.056 (0.023) | 0.055 (0.023) | 0.057 (0.024) | 0.063 (0.025) | 0.062 (0.025) |
| MDD | PE | -0.034 (0.033) | -0.034 (0.033) | -0.035 (0.033) | -0.039 (0.034) | -0.044 (0.034) | -0.043 (0.035) | -0.043 (0.036) |
|  | NEG | -0.026 (0.026) | -0.027 (0.026) | -0.029 (0.026) | -0.029 (0.026) | -0.029 (0.026) | -0.022 (0.026) | -0.028 (0.028) |
|  | DEP | -0.006 (0.032) | -0.002 (0.032) | 0.000 (0.032) | -0.004 (0.032) | -0.008 (0.033) | -0.013 (0.034) | -0.024 (0.035) |
|  | ANX | 0.008 (0.028) | 0.014 (0.028) | 0.015 (0.028) | 0.015 (0.029) | 0.016 (0.030) | 0.025 (0.031) | 0.027 (0.031) |
|  | GENERAL | 0.043 (0.023) | 0.044 (0.022) | 0.044 (0.023) | 0.045 (0.023) | 0.046 (0.023) | 0.047 (0.024) | 0.050 (0.025) |
| NEU | PE | -0.058 (0.033) | -0.052 (0.033) | -0.050 (0.034) | -0.052 (0.034) | -0.054 (0.034) | -0.060 (0.035) | -0.060 (0.038) |
|  | NEG | 0.001 (0.025) | 0.001 (0.025) | -0.001 (0.025) | 0.000 (0.025) | 0.003 (0.026) | 0.007 (0.028) | 0.009 (0.031) |
|  | DEP | -0.024 (0.030) | -0.017 (0.030) | -0.014 (0.031) | -0.010 (0.031) | -0.006 (0.031) | -0.002 (0.033) | -0.009 (0.036) |
|  | ANX | 0.052 (0.026) | 0.055 (0.026) | 0.054 (0.026) | 0.054 (0.026) | 0.057 (0.027) | 0.065 (0.028) | 0.067 (0.028) |
|  | GENERAL | 0.071 (0.022) | 0.073 (0.022) | 0.071 (0.022) | 0.071 (0.022) | 0.073 (0.022) | 0.075 (0.023) | 0.082 (0.024) |
| BIP | PE | -0.023 (0.033) | -0.031 (0.033) | -0.034 (0.034) | -0.038 (0.034) | -0.039 (0.034) | -0.036 (0.035) | -0.028 (0.038) |
|  | NEG | 0.033 (0.027) | 0.033 (0.027) | 0.035 (0.027) | 0.038 (0.028) | 0.039 (0.028) | 0.038 (0.029) | 0.038 (0.030) |
|  | DEP | 0.013 (0.033) | 0.013 (0.033) | 0.017 (0.033) | 0.019 (0.033) | 0.021 (0.034) | 0.025 (0.036) | 0.019 (0.038) |
|  | ANX | -0.015 (0.027) | -0.019 (0.027) | -0.021 (0.028) | -0.021 (0.028) | -0.023 (0.028) | -0.026 (0.029) | -0.024 (0.030) |
|  | GENERAL | -0.030 (0.023) | -0.030 (0.023) | -0.028 (0.023) | -0.024 (0.023) | -0.024 (0.023) | -0.026 (0.024) | -0.023 (0.025) |

Note: SCZ, schizophrenia; MDD, major depressive disorder; NEU, neuroticism; BIP, bipolar disorder; PE, psychotic experience; NEG, negative dimension; DEP, depression; ANX, anxiety; GENERAL, general psychopathology; SE, standard error. Inverse probability weighted refers to analyses including sampling weights for non-response, where the “% truncated” indicates whether the weights were truncated based on the 50^th^ percentile (2.48), 75^th^ percentile (3.02), 90^th^ percentile (3.95), 95^th^ percentile (5.33) and 99^th^ percentile (8.94).

^1^ Standardized estimate

Supplementary figure 1. Associations between psychopathology latent traits and polygenic risk scores (PRS) for psychiatric disorders


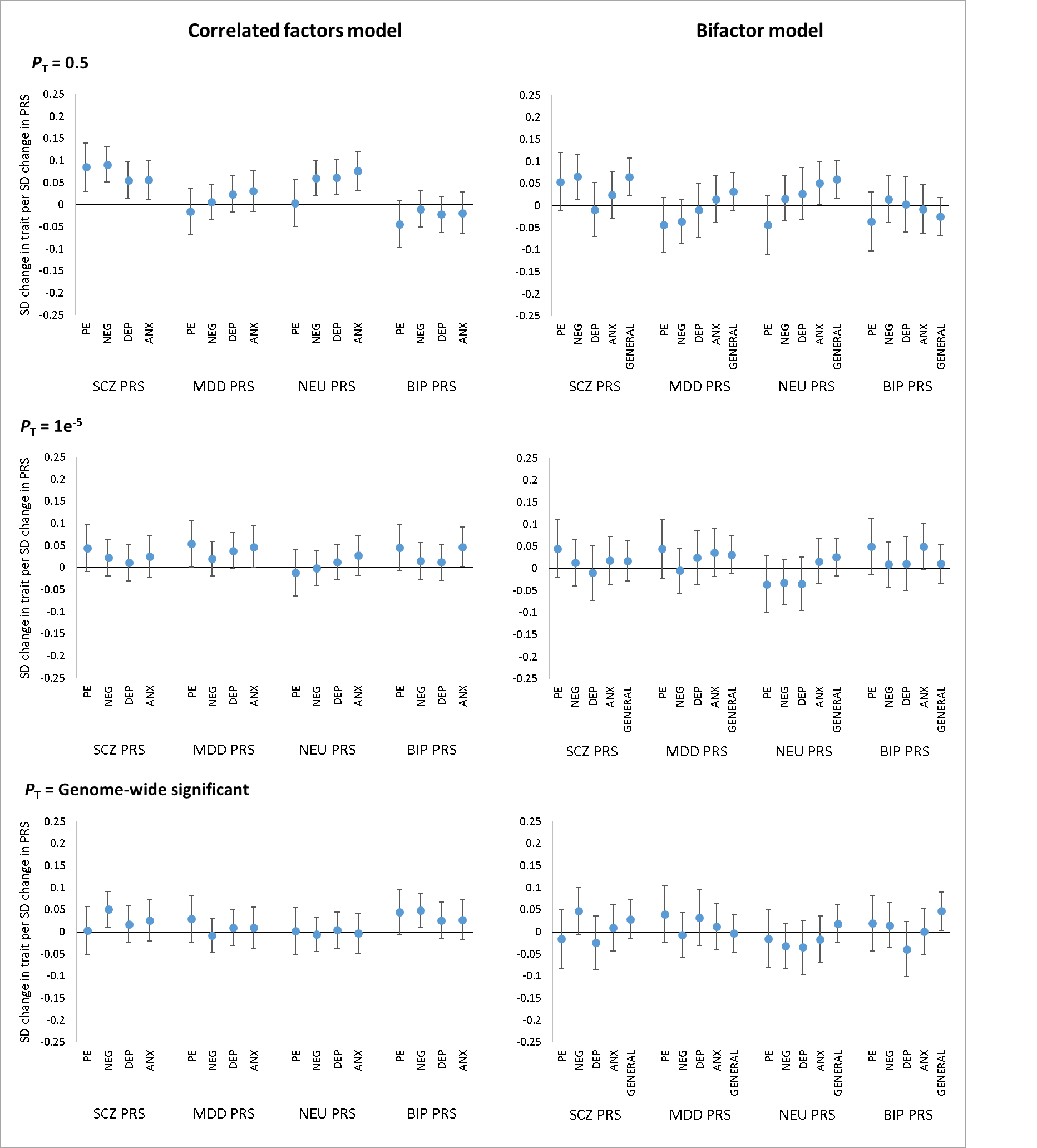


Associations are shown between latent traits for psychotic experiences (PE), negative dimension (NEG), depression (DEP), anxiety (ANX) and general psychopathology (GENERAL) generated using a correlated factors model (left-hand figures) and a bifactor model (right-hand figures) and polygenic risk scores (PRS) for schizophrenia (SCZ), major depressive disorder (MDD), neuroticism (NEU) and bipolar disorder (BIP) generated using lists of SNPs meeting 4 P-value thresholds (*P*_T_). Standard deviation (SD) changes in latent trait per SD change in PRS are shown with upper and lower 95% confidence intervals. Genome-wide significant *P*_T_ = polygenic score created from independent genome-wide significant SNPs as reported by discovery studies. N = 2863.
